# Ultrasound-Assisted Extraction of Polysaccharides from *Pleurotus ostreatus* By-Products: Box–Behnken Optimization and Low-Fat Cookies Formulation

**DOI:** 10.3390/foods15101764

**Published:** 2026-05-16

**Authors:** Patricia Bermúdez-Gómez, Vanessa Grifoll, Paula Bravo, Margarita Pérez-Clavijo

**Affiliations:** Mushroom Technological Research Center of La Rioja (CTICH), Carretera Calahorra, KM 4, 26560 Autol, La Rioja, Spain; microbiologia@ctich.com (V.G.); direccion@ctich.com (M.P.-C.)

**Keywords:** mushrooms by-products, *Pleurotus ostreatus*, Box–Behnken design, total dietary fiber, low-fat cookies, starch digestibility, glycemic index

## Abstract

Spent mushroom substrate (SMS), the main by-product of mushroom production, is rich in valuable compounds that could be recovered by ultrasound-assisted extraction (UAE) and exploited as fat-mimetic functional ingredients in food formulations. In this study, low-fat cookie prototypes were developed by incorporating a dietary fiber extract obtained from SMS using UAE. The extraction process was optimized following a Box–Behnken experimental design, identifying optimal conditions at a specific energy input of 200 J/mL, a particle size of 2 mm, and a solvent-to-solute ratio of 27%, yielding a dietary fiber recovery of 30.82%. The optimized SMS extract exhibited high oil-holding capacity (OHC) (1.39 g/g), emulsion stability (ES) (80%), and foaming capacity (FC) (83.55%). Four cookie formulations were evaluated, among which G1 (50% fat replacement) showed the best balance between consumer acceptability and an improved nutritional profile, characterized by higher protein (8.4 g/100 g), total dietary fiber (TDF) (7.10 g/100 g), and mineral contents. Notably, G1 cookies displayed a significant reduction in predicted glycemic index (pGI), decreasing from 83.84 in the control to 69.65. Overall, these results demonstrate that optimized SMS-derived dietary fiber is an effective functional ingredient for the development of low-fat, high-fiber, and reduced-glycemic cookies, contributing to the valorization of agro-industrial by-products within a circular economy framework.

## 1. Introduction

The global edible mushroom market has expanded significantly in recent years, with production expected to exceed 20 million tons annually by 2026 [1,2]. Among the most widely cultivated species is the oyster mushroom (*Pleurotus ostreatus*), an edible mushroom that grows on lignocellulosic materials originating from forest sources as well as from agricultural and food industry by-products [3]. During the production of fruiting bodies, considerable quantities of by-products are produced, mainly consisting of spent mushroom substrate (SMS), stems, and mushrooms without the required size or shape for commercial sale outs [2]. Spent mushroom substrate consists of fungal mycelia, nutrients, extracellular enzymes secreted by mushrooms, and a wide range of disintegrated lignocellulosic biomass, such as sawdust, corn cob, straw, and wood chips [4,5]. Consequently, SMS contains a range of valuable compounds, including trace elements (Fe, Ca, Zn, and Mg), cellulose, hemicellulose, lignin, and crude protein [5]. Approximately 5 kg of SMS is released for every 1 kg of fresh mushrooms produced, resulting in the generation of about 60 million tons of this by-product annually, posing a significant challenge for producers [2,6]. Traditionally, mushroom by-products are disposed of through spreading on farmland, incineration, open burning, landfilling, or composting with animal waste [4,7]. These practices result in environmental impacts, such as soil and water contamination [1,6]. Furthermore, this process results in the loss of substantial amounts of organically rich material with considerable nutritional value, posing both economic and environmental challenges [1,5,6]. However, in recent years, non-conventional uses of SMS have been explored, including its application in bioremediation, animal feed, biogas production, or enzyme extraction [1,5,8].

Hot water extraction followed by precipitation with alcohol is an extended technique used for water-soluble polysaccharide extraction [9]. However, these methods require long extraction times and high temperatures [10]. To mitigate these issues, green and novel extraction technologies, including ultrasound-assisted extraction (UAE), have emerged as an alternative for the recovery of bioactive compounds [8,10,11]. The improvement in extraction efficiency associated with ultrasound application is primarily attributed to acoustic cavitation phenomena generated in the solvent by ultrasonic waves [12]. These effects promote the disruption of cell wall structures and enhance mass transfer by accelerating diffusion across cellular membranes [12,13]. UAE has proven to be highly effective for the valorization of agro-industrial by-products through the isolation of a wide range of bioactive compounds, as it significantly reduces extraction time and solvent consumption [11,14]. The UAE has been investigated for the extraction of phenolic compounds and polysaccharides from spent mushroom substrate (SMS) derived from various edible mushroom species [8,11,15,16]. The extraction of polysaccharides from *Pleurotus ostreatus* SMS has been studied using an experimental design 2^3^ that explored the effect of temperature and sonication time on extraction efficiency [8].

Fats and oils play a crucial role in cookie quality, affecting both mechanical and sensory properties [17]. Fat enhances dough spread during baking, reduces fracture stress, increases cookie tenderness, and improves flavor and aroma [17,18]. However, the rising consumer interest in healthy diets and the link between high lipid intake and metabolic disorders have prompted the food industry to develop reduced-fat products and explore fat replacers [18,19]. Fat replacers can be carbohydrates or proteins that could imitate the functional and sensory properties of fat [18,19,20]. Proteins such as lupin and carbohydrates such as inulin or hydroxypropyl methylcellulose have been extensively studied and have demonstrated good properties and positive results in sensory analysis by reducing the fat content of cookies [18,19,20,21]. These compounds not only enable fat reduction but also provide health benefits, as dietary fiber is associated with improved gastrointestinal function and a reduced risk of metabolic and cardiovascular diseases [20,21,22]. The use of spent mushroom substrate (SMS) as a food ingredient has so far been mainly investigated as a wheat flour substitute in bread [3]. However, its high dietary fiber content makes SMS a promising candidate for fat and flour replacement [3,8]. In addition, several studies have reported that polysaccharides derived from SMS exhibit relevant bioactivities, including antibacterial, antitumor, antioxidant, and renoprotective effects [23,24,25,26].

To expand the current understanding of the UAE, this study investigates the impact of specific energy input, particle size, and solvent-to-solute ratio on the recovery of polysaccharides from *P. ostreatus* SMS. A Box–Behnken Design (BBD) was employed to optimize the extraction process, as it enables efficient evaluation of the interactions between variables while reducing the number of experimental runs and avoiding extreme experimental conditions. The functional role of the extract was tested as a fat and flour substitute in cookies, evaluating their physicochemical properties, sensory profile, and in vitro digestion kinetics to promote the integration of mushroom by-products into the circular food economy.

## 2. Methodology

### 2.1. Sample Preparation

The spent mushroom substrate (SMS) (wheat straw, corn husk, and urea) was defined as the residual matrix remaining after harvesting the fruiting bodies of *Pleurotus ostreatus* following two production flushes [24]. Cultivation was carried out at the CTICH facilities using the Alerpo strain, with spawn purchased from Amycel. Fresh SMS was dried in a dehydrator at 50 °C until a constant weight was reached (69.66% CH, 59.23% TDF: 26.80% celulosa, 15.41% hemicelulosa, 7.93% lignina, 17.00% Ash, 6.90% proteins). The dried mushroom by-product was then milled using an ultracentrifugal mill (ZM 200, Retsch™, Düsseldorf, Germany) equipped with four interchangeable sieves to obtain powders with particle sizes of 4 mm, 2 mm, 1 mm, and 0.25 mm. All powders were vacuum-packed and stored at room temperature in the dark.

### 2.2. Ultrasound-Assisted Extraction of Water-Soluble Polysaccharides (WSP)

The ultrasound-assisted extraction of water-soluble polysaccharides (WSP) from dried *Pleurotus ostreatus* spent mushroom substrate (SMS) powders was performed using an ultrasonic generator (UIP2000hdT, Hielscher Ultrasonics GmbH, Schwabach, Germany) with thermostatic temperature control. The equipment operated at a nominal power of 2000 W and 100% amplitude in all experiments. The extraction process was controlled based on the specific energy input (expressed as J/mL), while the total energy applied to each sample was expressed as Wh and calculated as Wh = (J/mL × volume (mL))/3600s. This approach was selected to ensure consistent treatment across samples, as the effective sonication time may vary depending on sample volume, viscosity, and temperature, affecting the actual power transmitted to the medium. By fixing the total energy input, all samples received an equivalent treatment regardless of process dynamics. For the Box–Behnken experimental design, extractions were performed at laboratory scale using a working volume of 500 mL. Subsequently, the optimized conditions were scaled up to a semi-pilot scale (20 L) to obtain sufficient material for further analysis. The use of specific energy input enabled consistent transfer of the extraction conditions between scales. The extraction temperature was continuously monitored and maintained between 70 and 80 °C throughout the process.

SMS powders were mixed with distilled water and subjected to ultrasound treatment in which specific energy input (200–500 J/mL), solvent-to-solute ratio (15–40%), and particle size (0.25–2.00 mm) were systematically varied. After sonication, the suspensions were filtered to remove solid residues, and the resulting filtrates were concentrated using a rotary evaporator (R-220 PRO, Büchi, Flawil, Switzerland). The concentrated extracts were then mixed with absolute ethanol (≥99.8%, PanReac AppliChem, Barcelona, Spain) at a 1:4 (*v*/*v*) ratio and left to stand at room temperature for 16 h to allow polysaccharide precipitation. The precipitates were collected by filtration, freeze-dried (Alpha 1–2 LD plus, Christ, Sigma Laborzentrifugen GmbH, Osterode am Harz, Germany), and milled. The percentage of polysaccharides yield (%) was also calculated following Equation (1).

(1) $$Yield\;(\%\;w/w)\;=\;\frac{Weight\;of\;dried\;crude\;extraction}{Weight\;of\;SMS\;powder}\;\times \;100$$

#### 2.2.1. Single-Factor Experiment Design

In this experiment, the following three variables were investigated: specific energy input (J/mL), particle size (mm), and solvent-to-solute ratio (%). Their variable effects on the WSP extraction yield were assessed. Each sample was processed following the polysaccharide extraction procedure described above.

#### 2.2.2. Optimization of Ultrasound-Assisted Extraction

The optimal conditions for ultrasound-assisted extraction were determined by response surface methodology (RSM) using a Box–Behnken Design (BBD). Through the single-factor experiment, the appropriate ranges of the independent variables, specific energy input (A), particle size (B), and solvent-to-solute ratio (C), were selected. Table 1 shows the range and center point values of the three independent variables. The BBD in experimental design consisted of 15 experimental runs, including 13 factorial points, and 3 replicates at the central point; each experiment was performed in triplicate. Experimental runs were randomized to minimize the effects of uncontrolled variability. The three variables at the three levels were coded as −1, 0, and +1.

The yield (%) of WSP and the total dietary fiber content (%) were chosen as the response or dependent variables (Y). Total dietary fiber (TDF) was analyzed by a standardized enzymatic-gravimetric method Megazyme Total Dietary Fiber Kit (K-TDFR, Megazyme, Wicklow, Ireland), according to the AOAC Official Method 991.43 [27].

### 2.3. Proximate Composition and Minerals Profile

All determinations were performed in triplicate and expressed as g/100 g of dry weight. Proximate compositions were estimated using methods based on the Association of Official Analytical Chemists [28]. Moisture content was calculated by difference in weight after drying in an ED 400 dryer (Binder GmbH, Tuttlingen, Germany) at 105 °C compared to the constant weight. Total ash was determined using a 10-PR/400 muffle furnace (Hobersal, Barcelona, Spain) at 550 ◦C after 5 h. Crude protein was determined using the Kjeldahl method using a Kjeltec System 2200 nitrogen distiller (FOSS IBERIA, Barcelona, Spain) and a digestion block (FOSS IBERIA, Barcelona, Spain). Crude fats were extracted following the Folch method described by Eggers and Schwudke [29]. The samples were briefly hydrolyzed with HCl (3 M) for 1 h at 80 ◦C in a LSB18 shaking bath (VWR International Eurolab, Barcelona, Spain). Finally, fat was extracted via dilution (1:20, *v*/*v*) with chloroform: methanol (2:1, *v*/*v*). Total carbohydrates were calculated by subtracting moisture, total fat, protein, and ash at 100%. The mineral content was determined using inductively coupled plasma-mass 165 spectrometry (ICP-MS) Shimadzu MS-2030 (Shimadzu, Kyoto, Japan). All analyses were performed in triplicate, and results were expressed as mg/100 g of flour.

### 2.4. pH, Water Activity and Instrumental Color

The pH of the optimized WSP extract was measured by mixing 5 g of the sample with 40 mL of ultrapure water. The pH was measured using a GLP 21 pH meter (Crison Instrument S.A., Barcelona, Spain) after 10 min of stirring. Water activity (a_w_) was determined using a Novasina 137 Thermoconstanter Sprint TH-500 (Pfäffikon, Switzerland) at 25 °C. The color was measured with a chroma meter CR-400 colorimeter (Konica Minolta, ITA Aquateknica S.A., Valencia, Spain) with illuminant D65, observer 2°, SCI mode, 8 mm aperture for illumination and measurement, based on the CIELab color space. The following color coordinates were determined: lightness (L*), redness (a* ± red-green), and yellowness (b* ± yellow-blue). From these coordinates, chroma (C*) was calculated as: C* = (a*^2^ + b*^2^)^1/2^ [30].

### 2.5. Techno-Functional Properties

Water-holding capacity (WHC) was determined by adding 10 mL of ultrapure water to 500 mg of the extract. Subsequently, the mix was stored at room temperature for 18 h. After being centrifuged (4780 rpm, 20 min), the supernatant was discarded, and the pellet was weighed [31]. The WHC of each sample was expressed as the weight of water held per gram of the corresponding sample (g of water/g sample). Oil-holding capacity (OHC) was determined by following the same procedure, replacing water with oil. The results were expressed as grams of oil retained per gram of sample (g oil/g sample). Water absorption capacity (WAC) was determined according to the method described by Beuchat [32]. Briefly, 1 g of extract was mixed with 10 mL of distilled water and agitated for 1 h. The mixture was then centrifuged at 4750 rpm for 30 min, the supernatant was discarded, and the pellet was weighed. The results were reported as g of water held by g of sample (g/g). For the emulsifying capacity (EC), 1 g of extract was mixed with 50 mL of distilled water and agitated for 30 min. Subsequently, 50 mL of sunflower oil was added, and the mixture was agitated for an additional 30 min to form the emulsion. Then, 10 mL of the emulsion was transferred to graduated centrifuge tubes and centrifuged at 3000 rpm for 5 min to allow phase separation. The volume of the emulsified layer was measured and used to calculate the emulsifying activity of the extract as the percentage of the emulsified layer relative to the total volume in the centrifuge tube [33]. Emulsion stability (ES) was evaluated using the emulsions prepared for the emulsifying activity assay. The centrifuge tubes containing the emulsions were heated at 80 °C for 30 min, cooled to room temperature, and then centrifuged at 3000 rpm for 5 min. Emulsion stability was expressed as the percentage of the emulsified layer remaining relative to the initial emulsion volume. Foaming capacity (FC) of the extract was determined following the method described by Coffmann and Garcia [34]. Briefly, 1 g of extract was mixed vigorously with 50 mL of distilled water for approximately 5 min to generate foam using a blender. The resulting foam was carefully transferred to a graduated cylinder, and its initial volume was recorded. Subsequently, the foam volume was measured after 30 s to assess its stability. These values were used to calculate the foaming capacity and foam stability of the extract.

### 2.6. Cookie Development

Cookies were developed by utilizing various commercially available raw ingredients: wheat flour (125 g), butter (62.5 g), sugar (40 g), vanillin (20 g), egg white (16.25 g), cocoa (14.25 g), yeast (5 g), baking powder (1.25 g) and salt (0.125 g) (Table 2). This recipe was used as the control (CT). The WSP extract was incorporated to partially replace butter at 50% (G1) and 75% (G3). In addition, two formulations combined the replacement of butter (50% or 75%) with a 10% substitution of wheat flour, corresponding to G2 and G4, respectively (Figure S1).

Cookie dough was prepared using an HM300 kneader (Kenwood Ltd., Havant, UK). Initially, sugar and eggs were creamed until homogeneous, followed by the incorporation of butter and, where applicable, the SMS extract. After achieving a creamy texture, cocoa powder, vanillin, and water were added. The remaining dry ingredients were then incorporated, and the mixture was kneaded for 5 min to ensure a consistent dough. The dough was rolled out to a uniform thickness of 5 mm and cut into round shapes (3 cm diameter; 16 g weight). The samples were stored at –18 °C for a maximum of 10 days, with all formulations within each batch subjected to the same storage time to ensure uniform processing conditions before baking. Finally, the cookies were baked directly from frozen in an HR-38N RM7 electric oven (Grunkel, Madrid, Spain) at 200 °C for 12 min.

### 2.7. Chemical and Physicochemical Characterization

For the nutritional analysis of the cookies, the contents of protein, fat, moisture, ash, fiber and mineral were determined following the procedures described in Section 2.2.2. and Section 2.3. The energy value (kcal/100 g) of the samples was calculated based on their macronutrient composition, using the Atwater general factors. Specifically, protein, available carbohydrates, and fat contents were multiplied by their respective conversion factors (4, 4, and 9 kcal/g, respectively), and the results were summed to obtain the total energy content. The values were expressed per 100 g of sample on a dry weight basis. Instrumental color was assessed as outlined in Section 2.4. Color differences (ΔE*) of each sample concerning to control sample was measured as follows: ΔE= (ΔL*^2^ + Δa*^2^ + Δb*^2^)^1/2^. All analyses were conducted in triplicate.

### 2.8. Sensory Analysis

The cookie control and prototypes were submitted to a panel of 85 people (aged between 19 and 65 years old). Untrained consumers were recruited among staff at the Mushroom Technological Research Center of La Rioja (CTICH) and among local inhabitants. All participants were fully informed about the nature and composition of the food products before their participation, and written informed consent was obtained from each panelist before the sensory tests were carried out. The sensory evaluation was conducted using cookie samples 1 h after baking. Coded samples of cookies (1 × 1 × 1 cm) with a random 3-digit number were given to the evaluation panel on white plates. Water was used to rinse the mouth before and after each sample test. The panelists were asked to score the following attributes on a nine-point hedonic scale, where 1 = dislike extremely, 5 = neither like nor dislike, 9 = like extremely: appearance, color, aroma, graininess, hardness, stickiness, sweet taste, bitter taste, and overall acceptability.

### 2.9. In Vitro Digestion: Starch and Predicted Glycemic Index

In vitro gastrointestinal digestion was performed following the harmonized INFOGEST protocol (V 2.0) [35]. Prior to initiating the digestion process, the cookie samples were ground and passed through a 510 μm mesh sieve to simulate the particle size typically resulting from mastication. In brief, 500 mg of milled cookies was mixed with 500 μL of distilled water to obtain a pasta with tomato pasta consistency. Then, simulated salivary fluid with salivary alpha-amylase (75 U/mL) (α-Amylase from human saliva, Type XIII-A, 940 U/mg protein) was added, followed by incubation in agitation at 37 °C and 60 rpm. After 2 min, 1.6 mL of simulated gastric fluid and HCl (1 M, 0.25 M) until pH 3.0 was incorporated into the oral samples to stop oral digestion. After that, 100 μL of porcine pepsin solution (2000 U/mL) (Sigma-Aldrich P7012) was added. Then the samples were incubated in the same conditions as the oral phase for 2 h. Finally, the gastric phase was mixed with 1.7 mL of simulated intestinal fluid, 8 μL of CaCl_2_ (3 M), 0.5 mL of bile (Bile bovine B3883-25G), and 1 mL of pancreatin (Sigma Pancreatin P7545 8 × USP specifications) (Sigma-Aldrich, St. Louis, MO, USA). The pH was adjusted to 7.0 with NaOH (1 M, 0.25 M), and the samples were incubated for 2 h at 37 °C and 60 rpm of agitation. Starch hydrolysis after in vitro gastrointestinal digestion was monitored in the oral phase (2 min), at the two points of the gastric phase (20 and 120 min), and at three points of the intestinal phase incubation (140, 210, and 240 min). A heat shock (100 °C, 5 min) was carried out to inactivate digestive enzymes in the gastric and intestinal phases, and a pH shift to 3 was carried out to inactivate digestive enzymes in the oral phase. For the respective five points studied, an individual in vitro digestion was performed. The simulated digestion of 6 different endpoints was achieved in triplicate for all the cookies. After the digestive enzymes were inactivated, the digested samples were centrifuged at 10,000 rpm for 10 min.

Starch content among gastrointestinal digestion was determined according to AOAC Official Method 996.11 using a total starch assay kit (Megazyme, Bray, Ireland). Briefly, two aliquots of 0.45 mL were taken from each digestive endpoint and mixed with sodium acetate (100 mM) plus ClCa2 (5 mM), pH 5.0 (dilution 1:4), and amyloglucosidase (20 μL; 60 U/mL) to complete the digestion of glucose disaccharides and oligosaccharides. The samples were incubated at 50 °C for 30 min. After that, the samples were diluted (1:10). Finally, aliquots of 50 μL digestive dilution were mixed with glucose oxidase/peroxidase reagent (GOPOD) (1.5 mL) and incubated for 30 min at 50 °C. Then, absorbance was measured at 510 nm. A blank was prepared by substituting samples with sodium acetate (100 mM) plus CaCl_2_ (5 mM), pH 5.0. Three glucose patterns (1 mg/mL) were included in each reaction. For each batch, an enzyme-free blank containing the sample was included to prevent overestimation of starch hydrolysis following in vitro gastrointestinal digestion. Equation (2) was used to calculate the percentage of starch after in vitro digestion.

(2) $$\%\mathrm{Starch}=\Delta A\times F\times \frac{\mathrm{VD}}{0.05}\times D\times \frac{1}{1000}\times \frac{100}{W}\times \frac{162}{180}$$

where

A = Absorbance sample;
F = factor to convert absorbance values to μg of D-glucose (100 μg of D-glucose divided by the GOPOD absorbance value for 100 μg of D-glucose);
VD = Digestion phase volume (mL);
D = Dilution factor;
W = Sample dry weight (mg);
162/180 = Factor to convert from free glucose, as determined, to anhydroglucose, as occurs in starch.

Finally, to assess the percentage of hydrolyzed starch, total starch content of the raw cookies was also determined. Predicted glycemic index (pGI) was calculated as the area under the curve (AUC) of each studied cookie formulation, using the first-order equation of the hydrolytic process (Equations (3) and (4)), and white bread as reference food. Equations (5) and (6) proposed by Goñi et al. were also used for calculating the pGI [36]. The concentrations obtained at 210 min were used as the final reaction time.

(3) $$C=C\infty (1-\;e^{-\mathrm{kt}})$$

(4) $$\mathrm{AUC}=C\infty \left(t\infty -t0\right)-(C\infty /k)\left[1-e^{-k(t\infty -t0)}\right]$$

(5) HI=AUCcookie/AUCwhite bread×100

(6) pGI=39.71+0.549 HI

where

C = % hydrolyzed starch;
C_∞_ = % hydrolyzed starch at final time;
k = kinetic reaction constant;
t_∞_ = final reaction time (140 min);
t_0_ = start reaction time;
HI = hydrolysis index;
AUC = area under the curve;
pGI = predicted glycemic index.

### 2.10. Statistical Analysis

All the results are presented as the mean ± standard error of the mean (SEM) of at least three independent, triplicate measurements. Data obtained for all the determinations, including hedonic punctuation in sensory analysis, were analyzed using one-way ANOVAs. Tukey’s post hoc test was applied for comparisons of means; differences were considered significant at *p* < 0.05. The extraction of total dietary fiber was optimized using a Box–Behnken design. The experimental data were fitted to a second-order polynomial model using Statgraphics (Centurion 19-X64). The fitting quality of the model was evaluated by Analysis of Variance (ANOVA), based on the coefficient of determination (R^2^) and Durbin-Watson (DW) statistics [37,38]. Finally, the predicted values and regression equations obtained from Statgraphics were used to construct the response surface plots in OriginPro 2026 (OriginLab Corporation, Northampton, MA, USA) for enhanced graphical visualization as well as the radial plot for sensorial evaluation. The applied polynomial model was Equation (7):

(7) $$Y=\beta_{0}+\sum_{i=1}^{k}\beta_{i}X_{i}+\sum_{i=1}^{k}\beta_{ii}X_{i}^{2}+\sum_{i<j}^{k}\beta_{ij}X_{i}X_{j}+\varepsilon$$

where Y is the dependent variable, β0 is the independent term, βi are the linear regression coefficients, βii are the quadratic regression coefficients, βij are the interaction regression coefficients, Xi or Xj are the independent variables or factors, and k is the number of independent variables or factors. To compare the experimental and theoretical values, a validation analysis was performed at 95% confidence level, where a *p*-value > 0.05 indicates no statistically significant difference.

## 3. Results and Discussion

### 3.1. Optimization of Ultrasound-Assisted Extraction

The Box–Behnken design was applied to evaluate and optimize the extraction of total dietary fiber from SMS powder as a function of three independent variables (specific energy input, particle size, and solvent-to-solute ratio), with a recognized effect on UAE efficiency [13,14]. The parameters considered during UAE fiber optimization were specific energy input (200–500 J/mL), particle size (0.25–2.00 mm), and solvent-to-solute ratio (15–40%). This range of studied variables was selected based on results from preliminary experiments (Figure S2) and previous studies on UAE for the extraction of polysaccharides from SMS in edible mushrooms [15,16]. Based on these results, a higher specific energy input (400 J/mL) increased extraction yield (19.21%) compared to 100 J/mL (17.53%) (*p* < 0.05), in agreement with previous reports [10,14]. At a constant volume and amplitude, as applied in this study, specific energy is directly related to sonication time. From 100 W to 400 W, fiber yield increased rapidly; however, a maximum yield was reached at 400 W, beyond which no further improvement was observed [10,14]. A similar trend has been previously reported for sonication time [39]. Regarding particle size, a higher extraction yield was observed as particle size decreased from 1 mm (18.72%) to 0.25 mm (21.85%). This behavior can be attributed to the increased surface area available for solvent contact, which enhances mass transfer [40].

**Figure 1:**
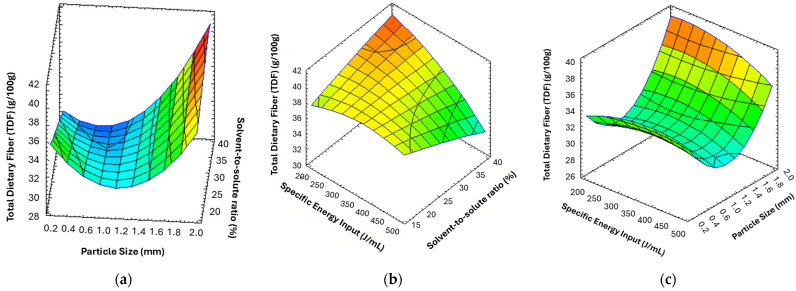
Response Surface plots showing the interactions between process parameters (**a**) Solvent-to-solute and Particle size; (**b**) Solvent-to-solute ratio and Specific energy input; (**c**) Particle size and Specific energy input.

Finally, to assess both the individual effects and interactions among the independent variables on the response, a second-order polynomial (quadratic) model was used. A total of 15 experimental runs were carried out, including three replicates at the center point to estimate the experimental error and assess the model reproducibility Although polysaccharide yield was also initially evaluated as a potential response variable, it was not selected for model development due to its poor fitting quality and low predictive ability (lower R^2^ values and non-significant model performance compared to TDF). Therefore, the experimental matrix and the corresponding TDF content are presented in Table 3. The TDF content varied significantly across the experimental domain (*p* < 0.05), ranging from 23.27% to 40.90%, indicating a strong dependence of the extraction efficiency on the selected process variables, as further illustrated by the response surface plots (Table 3, Figure 1). The highest TDF value (*p* < 0.05) was obtained at intermediate levels of specific energy input combined with the lowest level of particle size and the lowest level of solvent-to-solute (experiment 5). In contrast, the lowest TDF amount (*p* < 0.05) was observed under high specific energy input and solvent-to-solute ratio conditions, with an intermediate particle size (experiment 12). These results suggest significant interactions among the factors studied, as further confirmed by the fitted response surface model.

By applying multiple regression analysis on the experimental data, the response and test variables were found to correlate by the following second-order polynomial Equation (8):

Y = 28.3858 + 0.0742623 × A − 15.4129 × B + 0.0469339 × C − 0.0000516654 × A2 − 0.00884597 × A × B − 0.00119018 × A × C + 7.0195 × B2 + 0.193959 × B × C − 0.000965527 × C2
(8)

where Y is the Total Dietary Fiber, and A, B and C are the coded variables’ specific energy input, particle size and solvent-to-solute ratio, respectively.

Table 4 summarizes the results of the analysis of variance, goodness-of-fit, and the adequacy of the model. The model’s reliability was assessed using the Durbin–Watson (DW) statistics and a comparative analysis of the determination coefficients (R^2^). The R-value for Equation (8) was 0.8584, which was relatively high, indicating a close agreement between experimental and predicted values of the TDF amount. The adjusted R^2^ is a statistical measure used to evaluate the goodness-of-fit of the regression equation. The value R^2^ adjusted for Equation (8) was 0.8307, indicating that 83.07% of the total variation in the response could be attributed to the experimental variables studied. Furthermore, the R^2^ predicted for Equation (8) was 0.7862. The obtained DW value of 2.374 (*p* = 0.9010) confirms the absence of significant serial autocorrelation in the experimental data (*p* > 0.05). This result ensures that the variations in TDF content are due to the factors studied and not to systematic experimental errors, providing high reliability to the ANOVA results. The precision of the mathematical fit was further evidenced by the standard error of the estimate (1.9314) and the mean absolute error (MAE = 1.4912). These values, expressed in the same units as the response variable (%), indicate a high level of accuracy in the model’s predictions. The low MAE value suggests that the average deviation between the experimental and predicted total dietary fiber content is minimal, confirming the reliability of the Box–Behnken design for this extraction process.

The adequacy of the model was further justified through analysis of variance (ANOVA), as shown in Table 5. The significance of each coefficient was evaluated using the *p*-value. Variables are considered more significant when the F-value is higher, and the *p*-value is lower. Additionally, the *p*-value can be used to assess the strength of interactions between independent variables [9]. In this case, the independent variables (B and C), the interaction terms (AA, AB, AC, BB, and BC) significantly affected the content of TDF. Ultrasonic energy alone did not exert a significant main effect but showed negative interactions with the solvent-to-solute ratio (AC). High specific energy input, combined with an elevated solvent ratio, decreased TDF extraction by reducing the effect of cavitation intensity in the SMS powder [14]. Particle size exerted a dominant positive effect, particularly in its quadratic form (BB, *p* < 0.0001). These results differ from established mechanisms where reduced particle size enhances specific surface area, allowing ultrasonic cavitation to accelerate cell wall disruption [41]. However, excessive grinding may increase viscosity, limit cavitation intensity and hinder fiber solubilization [42]. Finally, the interaction between particle size and ratio was significantly positive (*p* = 0.0001), reinforcing the synergy between solvent availability and high specific surface area for enhanced mass transfer, aligning with previous reports of UAE of dietary fiber [43].

Based on the data obtained from Equation (8) using RSM, three-dimensional response surface plots (Figure 1) were generated to visualize the relationships between the responses and the processing variables, as well as the interactions between pairs of variables. These plots were constructed by representing the TDF content on the *Z*-axis as a function of the two processing variables that showed significant effects.

Figure 1a illustrates the three-dimensional response surface plot describing the combined effect of solvent-to-solute ratio and particle size on total dietary fiber (TDF) content. An overall increasing trend in TDF was observed with increasing particle size, particularly at higher solvent-to-solute ratios. Confirming the hypothesis of a possible limitation in cavitation intensity and fiber solubilization due to increased viscosity caused by excessive grinding [42]. At low particle sizes, TDF values remained comparatively low regardless of the solvent-to-solute ratio. In contrast, TDF content increased markedly with increasing particle size. The highest TDF values were obtained at the upper levels of both variables, indicating a significant interaction between particle size and solvent-to-solute ratio on TDF yield. Figure 1b shows the three-dimensional response surface plot at varying solvent-to-solute and specific energy input. The maximum extraction of polysaccharides was achieved under lower specific energy input and higher solvent-to-solute ratio conditions. Figure 1c shows the three-dimensional response surface plot across different specific energy inputs and particle sizes. The maximum extraction of polysaccharides was achieved at lower specific energy input and larger particle size.

The optimal extraction conditions obtained in the present study were specific energy input 200 J/mL, particle size 2 mm, and solvent-to-solute ratio 27%. Under these conditions, the response value predicted by the model was 39.60%.

### 3.2. Characterization of Optimized Dietary Fiber Extracts

The extraction conditions were validated by means of five independent extractions. The results of the characterization of this optimized extract in terms of chemical composition, physicochemical characteristics, and techno-functional properties are shown below.

#### 3.2.1. Proximate Composition of Dietary Fiber Extract

The proximate composition of the optimized SMS extract is presented in Table 6. The extraction process significantly altered the chemical profile of spent mushroom substrate. Specifically, the protein content increased from 6.9 g/100 g of dehydrated SMS to 14 g/100 g in the SMS extract. This behavior contrasts with previous reports on ultrasound-assisted extraction of polysaccharides from SMS, where a decrease in protein content after extraction was observed [15]. This discrepancy may be attributed to differences in ultrasound application, since that study employed an ultrasonic bath, which provides lower ultrasonic power compared to a probe system [14]. Consequently, the higher ultrasonic intensity applied in the present study may have promoted the solubilization of a greater amount of protein, since ultrasound-assisted extraction is inherently non-selective [14]. In addition, proteins could precipitate in the presence of ethanol [44]. Other studies have highlighted differences between purified and non-purified SMS polysaccharide extracts obtained using the Sevag method for deproteinization [45]. These studies reported not only a reduction in protein content but also a reduced antioxidant activity and phenolic compounds [45]. Considering these findings, together with the potential health and technological beneficial effects of protein–polysaccharide complexes, extract purification was intentionally avoided in the present study [45,46,47].

Regarding total dietary fiber, a significant reduction was observed in the optimized extract (30.82 g/100 g) compared to dehydrated SMS (59.23 g/100 g) (*p* < 0.05). It should be noted that the extraction process leads to the loss of insoluble dietary fiber, as the fraction not dissolved in the solvent is discarded during extraction. Consequently, the water-soluble polysaccharides recovered from SMS are mainly composed of soluble dietary fiber, particularly hemicelluloses [4,6,48]. In dehydrated SMS, the hemicellulose content accounted for 15.41%, supporting this hypothesis. In addition, ultrasound-assisted extraction may promote partial cellulose degradation into shorter chains, thereby increasing their solubility [41]. About total carbohydrates, a decrease was also observed, from 69.66% in the dehydrated SMS to 55.4% in the extract, suggesting a partial removal of the non-structural carbohydrate matrix during optimization. Similar carbohydrate contents have been reported in polysaccharide extracts obtained from *Pleurotus* SMS [15,45]. Finally, a notable increase in ash content was observed in the SMS extract (23.0 g/100 g) compared to the dehydrated SMS (17.0 g/100 g), indicating a concentration of mineral compounds. According to Regulation (EU) No 1924/2006, the extract could be considered high in calcium, magnesium, iron, manganese, zinc, and copper [49].

#### 3.2.2. Water Activity, pH, and Instrumental Color Parameters of Dietary Fiber Extract

The physicochemical parameters of the extract are observed in Table 7. The extract showed a pH of 5.60 ± 0.01 and a low water activity (a_w_) of 0.11 ± 0.01, which ensures a low risk of deterioration caused by microbial activity and enzymatic or non-enzymatic reactions, and therefore a long shelf-life for the dehydrated ingredient. The SMS extract exhibited a lightness (L*) value (67.13) that was higher than that reported for some dietary fiber extracts, but lower than values described for others [50,51]. Regarding redness (a*) and yellowness (b*), values of 4.47 and 21.46, respectively, were higher than those reported for soluble dietary fiber extracted from other agro-industrial by-products [50,51]. These color characteristics may be associated with the co-extraction of pigments during ultrasound-assisted optimization [14,45]. Overall, these instrumental color parameters should be carefully considered when incorporating the extract into food formulations, as they may induce noticeable color changes in the final product [50].

#### 3.2.3. Techno-Functional Properties of Dietary Fiber

The techno-functional properties of the optimized SMS extract are summarized in Table 8. Regarding hydration properties, the extract showed low WHC (0.24 g/g) and WAC (0.06 g/g). This could be due to SMS dietary fiber extract composition, which mainly consisted of soluble dietary fiber (SDF) or water-soluble polysaccharides with high solubility [43]. However, in this case, the extract did not fully solubilize in water, in contrast to what had been previously reported for other soluble fiber sources [43]. Although ultrasound applications may enhance water interactions by promoting the exposure of hydrophilic groups, such as hydroxyl and carboxyl groups on the SDF surface, excessive ultrasound treatment can also induce drastic structural changes [41,42,52]. These changes may disrupt polysaccharide intermolecular interactions, leading to a less porous structure and, consequently, reduced water retention capacity [41,42,51,52]. A similar trend was observed for OHC, which was relatively low (1.39 g/g) but comparable to values reported for other dietary fiber-rich by-products, indicating potential applicability as fat mimetics [30,41,43,50]. OHC reflects the oil-binding ability of the SDF extract and is closely associated with fiber surface structure, hydrophobicity, and charge density [41]. In this context, the OHC observed for the extract may be partially attributed to the presence of proteins containing hydrophobic amino acids [41,52].

Regarding emulsifying properties, EC refers to the ability of a substance to reduce interfacial tension between immiscible liquids, whereas ES describes its ability to maintain the emulsion and its resistance to rupture [53,54]. The extract showed a low EC value (5%), which may be associated with its relatively low oil-holding and water-holding capacities (OHC = 1.39 g/g; WHC = 0.24 g/g), suggesting a limited ability to rapidly adsorb and unfold at the oil–water interface during emulsion formation. However, once the emulsion is formed, the high ES (80%) can be explained by the presence of polysaccharide–protein complexes, which may improve interfacial film strength and provide steric stabilization at the oil–water interface [53]. In addition, SMS polysaccharides may increase the viscosity of the aqueous phase and delay droplet coalescence, further contributing to emulsion stability [55]. Therefore, the observed “low emulsifying activity–high stability” behavior can be attributed to a limited initial interfacial adsorption capacity, followed by strong stabilization of the formed emulsion through viscosity enhancement and interfacial structuring effects of polysaccharide–protein interactions. This surface activity was also reflected in the high foaming capacity (83.55%) of the extract. Therefore, the optimized SMS extract shows promising stability of high-fat foods, such as cookies [51,55].

### 3.3. Development and Characterization of Low-Fat Cookies by the Addition of Optimized Dietary Fiber Extract

After characterizing the composition and the physicochemical and techno-functional properties of the SMS extract, its application as a fat substitute in cookies was assessed. For this purpose, the nutritional profile, instrumental color, sensory properties, and digestibility were evaluated.

#### 3.3.1. Nutritional Analysis of Low-Fat Cookies

The chemical composition of the control and reformulated cookie prototypes is summarized in Table 9. Replacing butter and wheat flour with the optimized dietary fiber extract significantly altered the nutritional profile of the final products (*p* < 0.05). Specifically, formulations containing higher proportions of the extract (G3 and G4) showed increased moisture content compared to the control (*p* < 0.05), which may be attributed to the higher water addition required to facilitate dough handling in these formulations [56]. Regarding protein content, all reformulated cookies exhibited significantly higher values than the control (*p* < 0.05), with G4 showing the highest content (9.0 g/100 g) compared to 7.1 g/100 g in the control. This increase in protein content has not been commonly reported in cookies formulated with fat replacers based on complex carbohydrates [18,56]. However, similar enrichment in protein content has also been observed when wheat flour is partially replaced with polysaccharides derived from *Pleurotus* spp. [57]. As intended, a significant reduction in fat content was achieved in all formulations containing the SMS extract (6.1–10.0 g/100 g) compared to the control (18.0 g/100 g) (*p* < 0.05). The most pronounced reductions were observed in G3 and G4 (6.1 and 6.6 g/100 g, respectively), corresponding to a 75% butter replacement. Comparable fat contents to those of the control have been reported in previous studies using similar formulations [18,56,58]. Additionally, similar lipid reductions have been documented following the incorporation of inulin, polydextrose, and other complex carbohydrates [18,56,58].

Ash content increased progressively with increasing extract incorporation (*p* < 0.05), suggesting mineral enrichment derived from the SMS extract addition. Notably, calcium content in G1 (557.11 mg/100 g) was approximately tenfold higher than in the control (52.45 mg/100 g) (*p* < 0.05). Furthermore, doubling the extract concentration from G1 (31.25 mg/100 g) to G4 (59.4 mg/100 g) resulted in a proportional increase in calcium content (*p* < 0.05). Overall, all analyzed minerals increased significantly with extract addition, showing a concentration-dependent trend (*p* < 0.05). Similar mineral enrichment has been reported in cookies fortified with mushrooms and other agro-industrial by-products [58,59,60,61]. Total carbohydrate content decreased in formulations where wheat flour was partially replaced (G2 and G3) compared to the control, G1, and G4 (*p* < 0.05). In contrast, total dietary fiber content increased significantly, from 3.16 g/100 g in control to 8.33 g/100 g in G4 (*p* < 0.05), consistent with previous studies that incorporated *P. ostreatus* into cookies [62]. Based on these results, all formulations could be classified as “high in fiber” according to Regulation (EC) No 1924/2006 [49]. As a consequence of increased fiber content and reduced fat levels, all reformulated cookies exhibited lower energy values than the control. Overall, these results highlight the potential of SMS extract not only as a fat replacer but also as a functional ingredient that can enhance the nutritional profile of bakery products by increasing fiber, protein, and mineral contents.

#### 3.3.2. Instrumental Color of Low-Fat Cookies

The surface color of the cookies, expressed as L*, a*, b*, C*, and ΔE values, is presented in Table 9. The incorporation of the SMS extract significantly affected the optical properties of the cookies (*p* < 0.05). Specifically, a* and b* values decreased in all SMS extract–containing formulations compared to the control (*p* < 0.05), resulting in lower chroma (C*). These changes may be primarily associated with the intrinsic color of the added extract, which differs from that of the original ingredients and may contribute directly to the observed color modification [63,64]. In addition, the presence of proteins and other reactive compounds in the formulation could potentially contribute to color development during baking through Maillard reactions [60]. However, this effect cannot be confirmed in the present study due to the lack of amino acid composition data. The ΔE values obtained (6.61–9.06) indicate that the color differences between reformulated and control cookies were perceptible to the human eye, as ΔE values exceeded the threshold of 3 [65]. Among the formulations, G3 exhibited the smallest color difference relative to the control (*p* < 0.05), which may be related to its higher moisture content and lower extract incorporation compared to G4. Similar color changes have been previously reported when fat or wheat flour was replaced with mushroom fruiting bodies or other complex carbohydrates [47,60,61]. As color is a key factor influencing consumer acceptance of food products, a sensory evaluation was conducted to assess the impact of these color changes and potential alterations in other quality attributes resulting from fat and flour substitution [50].

#### 3.3.3. Sensory Analysis of Low-Fat Cookies

**Figure 2:**
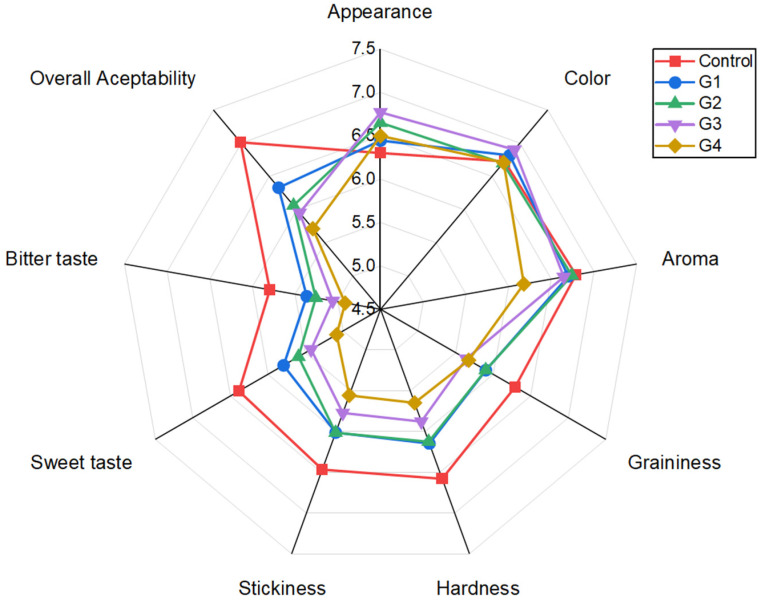
Radial plot of the sensory evaluation results of low-cookies formulated with the different concentrations of SMS extract compared to the control sample. G1: Cookie with 50% replace of butter; G2: Cookie with 50% replace of butter and 10% of wheat flour; G3: Cookie with 75% replace of butter; G4: Cookie with 75% replace of butter and 10% of wheat flour.

The sensory evaluation of all cookie formulations, including attributes such as appearance, graininess, hardness, stickiness, sweet taste, bitter taste, and overall acceptability, is presented in Figure 2. Despite the instrumental color differences, no significant differences in color liking were observed among samples (*p* > 0.05). Similarly, no differences were detected in appearance or graininess (*p* > 0.05). Comparable findings have been reported in studies where wheat flour substitution below 15% was achieved through the incorporation of mushroom powder or *Pleurotus*-derived fiber extracts [57,66]. Likewise, fat replacement using inulin or canned green pea purée did not negatively affect sensory perception in previous studies [58]. In contrast, G4, corresponding to the formulation with 75% fat and 10% wheat flour replacement and the highest extract concentration (23.76%), exhibited significant differences compared to the control in aroma, hardness, stickiness, and bitterness (*p* < 0.05), indicating a stronger influence of extract level and fat and flour reduction on sensory perception [18,19,57]. Similar effects have been reported when mushroom powder exceeded 15% or fiber extracts were incorporated at levels above 12% [57,67]. The SMS extract may contribute aromatic and flavor-active compounds due to the presence of volatile constituents in its composition [3,61]. The *Pleurotus ostreatus* SMS extract provides a complex profile of volatile compounds, in which eight-carbon (C8) derivatives predominate, characteristic of fungal aroma. Among these, 1-octen-3-ol is the most abundant compound and is responsible for earthy and fresh mushroom notes, together with 3-octanol and 3-octanone [68,69]. However, during baking, these native volatile compounds are attenuated due to evaporation and thermal degradation [70]. Simultaneously, the interaction between precursors present in the extract (such as amino acids and sugars like trehalose and mannitol) and the cookie matrix triggers Maillard reactions and Strecker degradation pathways. This leads to the formation of new compounds such as pyrazines (contributing roasted and nutty notes) and Strecker aldehydes (malty notes), which modify the original “raw mushroom” sensory profile towards more complex baked aromas [3,70]. It is well documented that one of the most critical challenges in fat replacement in bakery products is texture modification, often leading to increased hardness and less desirable mouthfeel [18,19]. In addition, high dietary fiber content has been associated with increased crispness and hardness [57].

Regarding sweetness, a progressive decrease was observed with increasing extract incorporation, with the lowest hedonic scores recorded for G4 (*p* < 0.05). These results are consistent with previous reports of sweetness reduction following 50% fat substitution [18]. However, no significant differences were found between the control and G1 (50% fat replacement) (*p* > 0.05). Among all formulations, G1 emerged as the most promising prototype, showing no significant differences compared to the control across all evaluated attributes (*p* > 0.05), including overall acceptability, which differed significantly between the control and all other formulations (*p* < 0.05), except for G1. In summary, formulation G1 enabled a substantial reduction in fat content while simultaneously increasing dietary fiber and protein without compromising the sensory acceptability of cookies [18,20].

#### 3.3.4. Kineticstic of Starch Digestion and Glycaemic Index of Low-Fat Cookies

**Figure 3:**
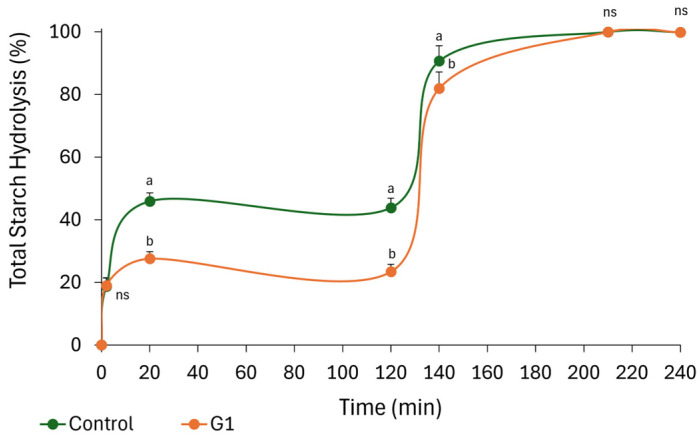
Total starch hydrolysis rate of cookies during gastrointestinal digestion. Results are reported as mean ± SEM (*n* = 6). Different letters indicated statistical difference when subjected to Tukey’s test (*p* < 0.05), ns: non statistically difference; G1: Cookie with 50% replacement of butter.

Based on the sensory evaluation results, only formulation G1 was selected for further analysis, as it exhibited the closest sensory profile to the control and the highest overall acceptability. In this context, the nutritional improvement of these cookies was further assessed by evaluating the *in vitro* digestibility of starch. As shown in Figure 3, starch hydrolysis in both the control and G1 began during the oral phase (2 min), driven by salivary α-amylase activity, with no significant differences observed between samples at this stage (*p* > 0.05). Starch hydrolysis continued during the gastric phase, with the first significant difference between samples observed after 20 min (*p* < 0.05), with hydrolysis values of 45.93% for the control and 27.57% for G1. Subsequently, starch hydrolysis remained relatively unchanged in both samples until the end of the gastric phase at 120 min (*p* > 0.05).

Although the contribution of salivary α-amylase during gastric digestion has traditionally been ignored due to the short contact with the food and the pH shift from 6.9 to approximately 3, residual enzymatic activity during this phase has been reported [71]. Based on *in vitro* digestion kinetics, starch can be classified into rapidly digestible starch (RDS), slowly digestible starch (SDS), and resistant starch (RS) fractions [72]. The RDS fraction corresponds to the starch hydrolyzed within the first 20 min of digestion [73]. Accordingly, replacing 50% of fat with the SMS dietary fiber extract significantly reduced RDS content compared to the control (*p* < 0.05). Similar reductions in RDS have been previously reported following the incorporation of dietary fiber-rich oyster mushroom or fibers derived from other agro-industrial by-products into cookies [57,74,75,76]. The reduced starch hydrolysis observed in the reformulated cookies compared to the control may be attributed to the high dietary fiber content of the G1 cookies (6.64 g/100 g). Dietary fiber can hinder enzyme–substrate interactions through physical encapsulation of starch granules or by increasing digesta viscosity [60,61,74,76]. In addition, fat content has been reported to play a key role in starch organization within cookie dough, with significant morphological differences observed when fat replacement exceeds 30% [20,21,77].

Upon initiation of the intestinal phase, marked by the addition of pancreatic α-amylase, both samples exhibited a hydrolysis peak at 140 min. In the control, this peak remained stable until the end of the digestion assay at 240 min, with no significant differences observed between 140, 210, and 240 min (*p* > 0.05). In contrast, starch hydrolysis in G1 did not stabilize until 210 min, with no significant differences between 210 and 240 min (*p* > 0.05). As shown in Table 10, although total starch content did not differ between samples (*p* > 0.05), the proportion of starch hydrolyzed at 140 min (SH_140_) was significantly lower in G1 (81.97%) than in the control (90.70%) (*p* < 0.05). These results further confirm the inhibitory effect of SMS extract incorporation and fat reduction on starch hydrolysis. Consistently, the kinetic constant (k) decreased significantly from 0.03255 in the control to 0.01454 in G1 (*p* < 0.05), indicating slower starch digestion kinetics in the reformulated cookies. The glycemic index (GI) is a nutritional parameter used to classify carbohydrate-rich foods according to their potential to increase postprandial blood glucose levels [78]. Beyond its clinical relevance, GI has been associated with the risk of developing metabolic and cardiovascular diseases [71,73,78]. Since direct measurement of GI in human subjects is time-consuming, invasive, labor-intensive, and costly, *in vitro* methodologies have been developed as a faster and more accessible alternative to predict *in vivo* glycemic (pGI) response [73,78]. In the present study, *in vitro* GI estimation was applied to assess the potential of the SMS extract to modulate the glycemic response of cookies. The area under the curve (AUC) and hydrolysis index (HI) values required for pGI calculation are presented in Table 10.

The physiological impact of dietary fiber incorporation and fat reduction on starch digestion was reflected in a significantly lower AUC_140_ for G1 (6446.24) compared to the control (9940.52) (*p* < 0.05). Accordingly, both HI and pGI values were significantly reduced in G1 relative to the control (*p* < 0.05). Based on GI classification criteria, foods can be categorized as low GI (≤54), medium GI (>55 to <70), or high GI (≥70) [72]. Under this classification, G1 (69.65) lies at the upper limit of the medium-GI range and could also be considered borderline high GI. Nevertheless, the pGI value of the control sample was markedly higher (85.88) than that of the cookie enriched with dietary fiber extract (G1), indicating a clear reduction in glycemic response (*p* < 0.05). High dietary fiber content is commonly associated with reduced pGI values, and similar reductions have been reported in fiber-enriched cookies compared with conventional formulations [75,76]. Overall, these findings highlight the potential of dietary fiber extracted from SMS to reduce the glycemic impact of cookies while maintaining sensory acceptability comparable to that of conventional products. However, a limitation of the present study is that the formulation did not achieve a low or clearly medium GI product, suggesting that further optimization could be explored to enhance pGI reduction while preserving consumer acceptance.

## 4. Conclusions

Spent mushroom substrate is a promising source of dietary fiber that can be used as a partial fat replacer in cookie production. The total dietary fiber content of the extract, optimized using response surface methodology (RSM) based on a Box–Behnken design, was 30.82%. The chemical and techno-functional characterization of the extract also revealed a high content of proteins and minerals, which provided suitable emulsifying and oil-holding properties. The incorporation of the optimized extract to replace 50% of fat in cookies (G1) resulted in a significantly higher content of protein, mineral, and total dietary fiber, reaching levels that allow them to be labeled as “high in fiber” in accordance with European Regulation (EC) No 1924/2006. In addition, G1 exhibited a significant reduction in the predicted glycemic index, which can be attributed to the high dietary fiber content and its ability to form a physical barrier that limits enzymatic access to starch. Importantly, the G1 prototype achieved an overall sensory acceptability comparable to that of the control formulation prepared with a traditional recipe. This result indicates an effective balance between the health benefits of fat reduction and fiber enrichment and the palatability expected in bakery products. Overall, these findings highlight the potential of SMS dietary fiber extract, obtained using an environmentally friendly technique such as ultrasound-assisted extraction, not only as a strategy for the valorization of an agro-industrial by-product but also as a promising tool for the food industry to develop cookies with an improved nutritional profile. Future research should address the long-term storage stability of these products and the in vivo validation of their glycemic response.

## Figures and Tables

**Table 1:** Levels and codes employed in the present study for the construction of Box–Behnken Design (BBD).

Variables	Codes	Levels		
		−1	0	+1
Specific energy input (J/mL)	A	200	350	500
Particle size (mm)	B	0.25	1.00	2.00
Solvent-to-solute ratio (%)	C	15	27.50	40

**Table 2:** Formulation of control cookies and low-fat prototypes by the addition of SMS extract.

Ingredients (g/250 g)	CT	G1	G2	G3	G4
Wheat flour	125	125	112.5	125	112.5
Butter	62.50	31.25	31.25	15.60	15.60
Sugar	40	40	40	40	40
Extract	-	31.25	43.75	46.90	59.40
Vanilla	20	20	20	20	20
Egg	16.25	16.25	16.25	16.25	16.25
Cocoa	14.25	14.25	14.25	14.25	14.25
Water	-	7.5	10	15	25
Yeast	5	5	5	5	5
Baking soda	1.25	1.25	1.25	1.25	1.25
Salt	0.125	0.125	0.125	0.125	0.125

CT: Control; G1: Cookie with 50% replacement of butter; G2: Cookie with 50% replacement of butter and 10% of wheat flour; G3: Cookie with 75% replacement of butter; G4: Cookie with 75% replacement of butter and 10% of wheat flour.

**Table 3:** Experimental matrix of the Box–Behnken design for the extraction of polysaccharides from SMS powder. The values correspond to the average of three independent trials ± standard error of the mean (SEM).

Experiments	A	B	C	TDF (%)
1	200	0.25	27.5	29.87 ^d^ ± 0.25
2	200	2.00	27.5	39.71 ^a^ ± 0.65
3	500	0.25	27.5	33.84 ^c^ ± 0.35
4	500	2.00	27.5	38.28 ^ab^ ± 0.20
5	350	0.25	15	40.90 ^a^ ± 0.53
6	350	2.00	15	38.26 ^ab^ ± 0.59
7	350	0.25	40	29.95 ^d^ ± 1.18
8	350	2.00	40	36.42 ^bc^ ± 0.14
9	200	1.00	15	31.67 ^cd^ ± 0.65
10	500	1.00	15	32.23 ^cd^ ± 0.11
11	200	1.00	40	31.60 ^cd^ ± 0.33
12	500	1.00	40	23.27 ^e^ ± 0.19
13	350	1.00	27.5	31.10 ^cd^ ± 0.70
14	350	1.00	27.5	31.51 ^cd^ ± 1.07
15	350	1.00	27.5	30.72 ^d^ ± 0.35

A: specific energy input; B: Particle size; C: solvent-to-solute ratio; TDF: total dietary fiber. Mean values within the same column followed by different superscript letters (a–d) are significantly different when subjected to Tukey’s test (*p* < 0.05).

**Table 4:** Statistical parameters for fitted model validation.

Statistic	Value
R-squared (R^2^)	85.84%
Adjusted R-squared	83.07%
Predicted R-squared	78.62%
Durbin–Watson statistic (*p*-value)	2.37 (0.9010)
Standard Error of the Estimate	1.9314
Mean Absolute Error (MAE)	1.4912

**Table 5:** Analysis of Variance (ANOVA) for the quadratic model of Total Dietary Fiber (TDF) extraction from SMS *Pleurotus ostreatus*.

Variables	Estimated Effect (Pred)	Standard Error	Estimated Effect (Adj)	Sum of Square	Df	Mean Sum of Squares	F-Value	*p*-Value
A: Specific energy input	−1.3755	0.8871	0.0432	14.4453	1	14.4453	3.87	0.0551
B: Particle size	4.5829	0.8833	−18.4114	161.728	1	161.728	43.36	0.0000 *
C: LSR	−5.1132	0.9098	−0.3049	189.739	1	189.739	50.87	0.0000 *
AA	−0.3259	1.3354	−0.000007	18.2239	1	18.2239	4.89	0.0321 *
AB	−2.3221	1.2195	−0.0088	21.7796	1	21.7796	5.84	0.0197 *
AC	−4.4632	1.2719	−0.0012	73.9783	1	73.9783	19.83	0.0001 *
BB	12.7893	1.3663	8.3522	371.749	1	371.749	99.66	0.0000 *
BC	4.2429	1.2721	0.1940	66.8316	1	66.8316	17.92	0.0001 *
CC	1.6973	1.3369	0.0054	0.3080	1	0.3080	0.08	0.7758
Total Error	N/A	N/A	N/A	171.588	46	3.7302	N/A	N/A
Total (corr.)	N/A	N/A	N/A	1211.90	55	N/A	N/A	N/A

* Stadistically significance (*p* < 0.05). LSR: Solvent-to-solute ratio; Df: Degree of freedom; N/A: Not Applicable.

**Table 6:** Proximate composition of optimized dietary fiber extract.

Sample	Protein	Fat	CH	TDF	Ash	Ca	Mg	Fe	Mn	Zn	Cu
SMS extract	14.0 ±0.11	1.40 ±0.06	55.4 ±0.56	30.82 ±0.52	23.0 ±0.23	4211.8 ± 5.13	663.6 ± 0.01	66.10 ± 0.43	52.00 ± 0.68	20.30 ± 0.33	2.27 ± 0.02

Results are reported as mean ± SEM (*n* = 3). Results of protein, fat, CH, TDF, and ash are expressed as g/100 g of dried weight. Results of minerals are expressed as mg/100 g of dried weight. CH: carbohydrates; TDF: total dietary fiber; Ca: calcium; Mg: magnesium; Fe: iron; Mn: manganese; Zn: zinc; Cu: copper.

**Table 7:** Physicochemical parameters of dietary fiber extract.

Sample	pH	a_w_	L*	a*	b*
SMS extract	5.60 ± 0.01	0.11 ± 0.01	67.13 ± 0.33	4.47 ± 0.15	21.46 ± 0.43

Results are reported as mean ± SEM (*n* = 3). a_w_: water activity; L*: lightness; a*: redness; b*: yellowness.

**Table 8:** Techno- functional properties of optimized dietary fiber extract.

Sample	WHC (g/g)	OHC (g/g)	WAC (g/g)	EC (%)	ES (%)	FC (%)
SMS extract	0.24 ± 0.08	1.39 ± 0.01	0.06 ± 0.00	5.00 ± 0.00	80.00 ± 0.00	83.55 ± 6.94

Results are reported as mean ± SEM (*n* = 3). WHC: Water-holding capacity; OHC: Oil-holding capacity; WAC: Water absorption capacity; EC: Emulsifying capacity; ES: Emulsion stability; FC: Foaming capacity.

**Table 9:** Nutritional composition and color of control and prototypes of low-fat cookies by the addition of optimized dietary fiber extract.

Parameters	Control	G1	G2	G3	G4
Moisture	14.0 ^c^ ± 0.15	14.0 ^c^ ± 0.16	15.0 ^b^ ± 0.20	16.0 ^a^ ± 0.10	16.0 ^a^ ± 0.23
Protein	7.10 ^c^ ± 0.13	8.40 ^b^ ± 0.11	8.50 ^ab^ ± 0.10	8.80 ^ab^ ± 0.15	9.00 ^a^ ± 0.12
Fat	18.0 ^a^ ± 0.20	9.2 ^b^ ± 0.25	10.0 ^b^ ± 0.30	6.1 ^c^ ± 0.09	6.6 ^c^ ± 0.08
Ash	1.90 ^e^ ± 0.01	4.4 ^d^ ± 0.02	5.70 ^c^ ± 0.02	5.8 ^b^ ± 0.01	6.8 ^a^ ± 0.01
CH	55.7 ^a^ ± 0.30	56.90 ^a^ ± 0.35	52.50 ^b^ ± 0.25	56.20 ^a^ ± 0.30	53.70 ^b^ ± 0.27
Energy (Kcal/100 g)	418.88 ^a^ ± 2.35	358.72 ^b^ ± 1.95	351.24 ^b^ ± 2.12	329.1 ^c^ ± 1.07	327.54 ^c^ ± 1.25
TDF	3.16 ^d^ ± 0.10	6.64 ^c^ ± 0.14	7.78 ^b^ ± 0.09	7.40 ^b^ ± 0.12	8.33 ^a^ ± 0.13
Ca	52.45 ^e^ ± 0.32	557.11 ^d^ ± 3.35	747.51 ^c^ ± 4.85	872.12 ^b^ ± 5.56	1000.0 ^a^ ± 6.69
Mg	48.26 ^e^ ± 0.25	130.5 ^d^ ± 1.25	161.55 ^c^ ± 1.35	170.76 ^b^ ± 1.15	199.84 ^a^ ± 1.89
Fe	2.72 ^d^ ± 0.06	7.94 ^c^ ± 0.12	9.69 ^c^ ± 0.36	17.8 ^a^ ± 0.65	12.2 ^b^ ± 0.49
Mn	0.76 ^c^ ± 0.09	5.62 ^b^ ± 0.15	7.13 ^ab^ ± 0.89	8.27 ^ab^ ± 0.98	8.78 ^a^ ± 0.58
Zn	1.1 ^c^ ± 0.08	3.73 ^b^ ± 0.06	4.6 ^ab^ ± 0.45	5.28 ^a^ ± 0.24	5.48 ^a^ ± 0.49
Cu	0.33 ^b^ ± 0.01	0.59 ^a^ ± 0.02	0.69 ^a^ ± 0.01	0.72 ^a^ ± 0.05	0.81 ^a^ ± 0.09
L*	28.82 ^a^ ± 0.16	26.54 ^b^ ± 0.30	28.94 ^a^ ± 0.25	27.22 ^b^ ± 0.25	29.23 ^a^ ± 0.19
a*	11.39 ^a^ ± 0.06	7.16 ^c^ ± 0.10	6.22 ^d^ ± 0.14	7.75 ^b^ ± 0.10	6.91 ^c^ ± 0.22
b*	10.54 ^a^ ± 0.16	3.67 ^c^ ± 0.09	3.13 ^c^ ± 0.18	5.29 ^b^ ± 0.17	3.26 ^c^ ± 0.28
C*	15.52 ^a^ ± 0.11	8.04 ^c^ ± 0.13	6.96 ^d^ ± 0.21	9.39 ^b^ ± 0.18	7.65 ^cd^ ± 0.30
ΔE	-	8.41 ^a^ ± 0.14	9.06 ^a^ ± 0.23	6.61 ^b^ ± 0.18	8.58 ^a^ ± 0.33

Results are reported as mean ± SEM (*n* = 3). Results of protein, fat, CH, TDF, and ash are expressed as g/100 g of dried weight. Results of minerals are expressed as mg/100 g of dried weight. Mean values within the same raw followed by different superscript letters (a–e) are significantly different when subjected to Tukey’s test (*p* < 0.05). G1: Cookie with 50% replace of butter; G2: Cookie with 50% replace of butter and 10% of wheat flour; G3: Cookie with 75% replace of butter; G4: Cookie with 75% replace of butter and 10% of wheat flour; CH: Carbohydrate; TDF: Total dietary fiber; Ca: calcium; Mg: magnesium; Fe: iron; Mn: manganese; Zn: zinc; Cu: copper; L*: Lightness; a*: redness; b*: yellowness; C*: chroma; ΔE: color difference.

**Table 10:** Starch digestion parameters of fat low-cookies formulated with a 50% fat reduction and the addition of optimized dietary fiber extract (G1).

Samples	TS (%)	SH_140_ (%)	k_140_	AUC_140_	HI_140_	pGI_140_
Control	29.16 ^b^ ± 1.32	90.70 ^a^ ± 3.06	0.03255 ^a^ ± 0.0001	9940.52 ^a^ ± 498.03	84.10 ^b^ ± 4.21	85.88 ^b^ ± 2.31
G1	33.70 ^b^ ± 2.18	81.97 ^b^ ± 1.74	0.01454 ^b^ ± 0.0006	6446.24 ^b^ ± 117.28	54.54 ^c^ ± 0.99	69.65 ^c^ ± 0.54
Bread	46.54 ^a^ ± 0.07	85.69 ^ab^ ± 0.94	0.03180 ^a^ ± 0.0001	9332.73 ^a^ ± 124.14	100.00 ^a^ ± 1.33	94.61 ^a^ ± 0.73

TS: Total Starch; SH_140_: total starch hydrolyzed at 140 min; *k*: kinetic constant; AUC: area under the curve; HI: hydrolysis index; pGI: predicted glycemic index. Mean values within the same column followed by different superscript letters (a–c) are significantly different when subjected to Tukey’s test (*p* < 0.05).

## Data Availability

The original contributions presented in this study are included in the article/Supplementary Materials. Further inquiries can be directed to the corresponding author.

## Supplementary Materials

The following supporting information can be downloaded at: https://www.mdpi.com/article/10.3390/foods15101764/s1, Figure S1: Low-fat cookies by the addition of SMS dietary fiber extract. CT: Control; G1: Cookie with 50% replacement of butter; G2: Cookie with 50% replacement of butter and 10% of wheat flour; G3: Cookie with 75% replace of butter; G4: Cookie with 75% replacement of butter and 10% of wheat flour.; Figure S2: Preliminary experiments on dietary fiber extraction from SMS of *Pleurotus ostreatus.* (**a**) Assessment of specific energy input (J/mL) vs extraction yield (%), (**b**) Assessment of particle size vs extraction yield (%).
