# Supplementary material for: Ultrasound-Assisted Extraction of Polysaccharides from Pleurotus ostreatus By-Products: Box–Behnken Optimization and Low-Fat Cookies Formulation
Source: Foods. 2026 May 16;15(10):1764. doi: 10.3390/foods15101764 (PMC13205208; doi:10.3390/foods15101764)
Supplement: Supplementary file 1 [file foods-15-01764-s001.zip › foods-4254419-supplementary.pdf]

Supplementary Materials

Ultrasound-Assisted Extraction of Polysaccharides from *Pleurotus ostreatus* By-Products: Box–Behnken Optimization and Low-Fat Cookies Formulation

P. Bermúdez-Gómez, V. Grifoll, P. Bravo, M. Pérez-Clavijo

Centro Tecnológico de Investigación del Champiñón en La Rioja (CTICH),
Carretera Calahorra, KM 4, 26560 Autol, La Rioja, Spain.

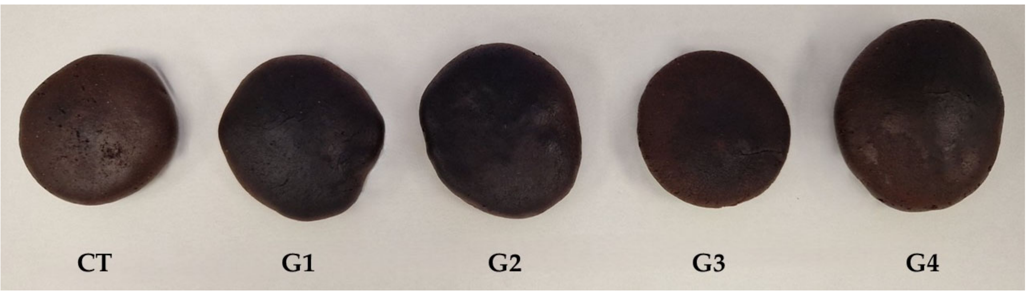

Figure S1. Low-fat cookies by the addition of SMS dietary fiber extract. CT: Control; G1: Cookie with 50% replacement of butter; G2: Cookie with 50% replacement of butter and 10% of wheat flour; G3: Cookie with 75% replace of butter; G4: Cookie with 75% replacement of butter and 10% of wheat flour.

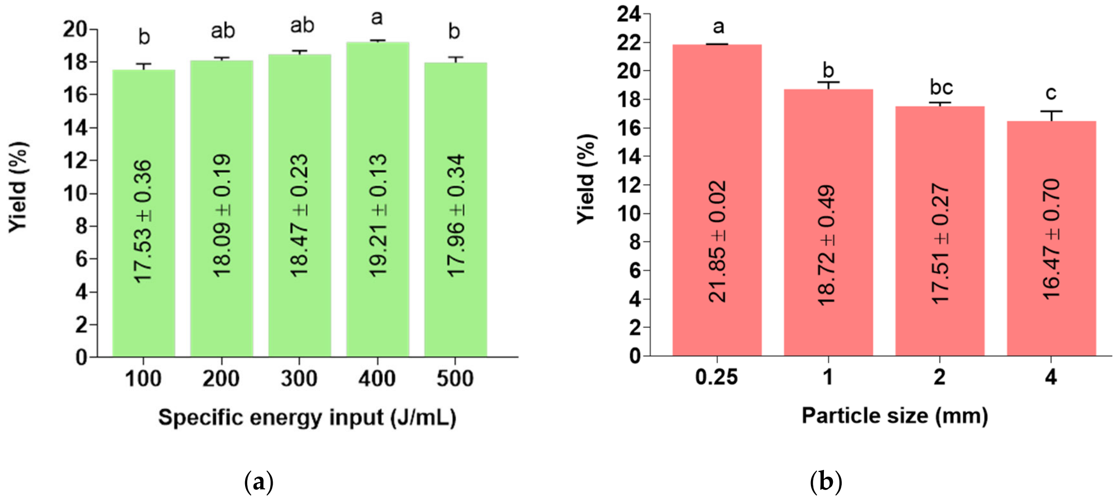

Figure S2. Preliminary experiments on dietary fiber extraction from SMS of *Pleurotus ostreatus*. (**a**) Assessment of specific energy input (J/mL) vs extraction yield (%), (**b**) Assessment of particle size vs extraction yield (%).
